# Alterations in Activation, Cytotoxic Capacity and Trafficking Profile of Peripheral CD8 T Cells in Young Adult Binge Drinkers

**DOI:** 10.1371/journal.pone.0132521

**Published:** 2015-07-07

**Authors:** José Luis Zaldivar Fujigaki, América Guadalupe Arroyo Valerio, Juan Carlos López Alvarenga, Esperanza Gabriela Gutiérrez Reyes, David Kershenobich, Joselin Hernández Ruiz

**Affiliations:** 1 Laboratory of Liver, Pancreas and Motility, Department of Experimental Medicine, Faculty of Medicine, Universidad Nacional Autónoma de México, Mexico City, Mexico; 2 Dirección de Investigación, Hospital General de México "Dr. Eduardo Liceaga", Mexico City, Mexico; 3 Instituto Nacional de Ciencias Médicas y Nutrición "Salvador Zubirán", Mexico City, Mexico; Oregon Health and Science University, UNITED STATES

## Abstract

**Background:**

Excess of alcohol consumption is a public health problem and has documented effects on the immune system of humans and animals. Animal and *in vitro* studies suggest that alcohol abuse changes CD8 T cell (CD8) characteristics, however it remains unknown if the CD8 profile of binge drinkers is different in terms of activation, trafficking and cytotoxic capacity.

**Aim:**

To analyze the peripheral CD8 cytotoxic capacity, activation and trafficking phenotypic profile of Mexican young adults with regard to alcohol consumption pattern.

**Methods:**

55 Mexican young adults were stratified as Light (20), Intermediate (18) or Binge drinkers (17) according to their reported alcohol consumption pattern. Blood samples were obtained and hematic biometry and liver enzyme analysis were performed. Peripheral CD8 profile was established by expression of Granzyme B (GB), CD137, CD127, CD69, TLR4, PD1, CCR2, CCR4, CCR5 and CXCR4 by FACS. Data was analyzed by ANOVA, posthoc DMS and Tamhane, and principal component analysis (PCA) with varimax rotation, p<0.05.

**Results:**

The Binge drinking group showed increased γGT together with increased expression of CD69 and reduced expression of TLR4, PD1, CCR2 and CXCR4 in peripheral CD8 cells. Other parameters were also specific to Binge drinkers. PCA established 3 factors associated with alcohol consumption: “Early Activation” represented by CD69 and TLR4 expression in the CD8 population; “Effector Activation” by CD69 expression in CD8 CD127^+^CD137^+^ and CD8 CD25^+^ CD137^+^; and Trafficking by CXCR4 expression on total CD8 and CD8 GB^+^CXCR4^+^, and CCR2 expression on total CD8. Binge drinking pattern showed low expression of Early Activation and Trafficking factors while Light drinking pattern exhibited high expression of Effector Activation factor.

**Conclusions:**

Alcohol consumption affects the immune phenotype of CD8 cells since binge drinking pattern was found to be associated with high CD69 and low TLR4, CXCR4 and CCR2 expression, which suggest recent activation, decreased sensitivity to LPS and lower migration capacity in response to chemokines SDF-1 and MCP-1. These results indicate that a binge-drinking pattern of alcohol consumption may induce an altered immune profile that could be related with liver damage and the increased susceptibility to infection reported to this behavior.

## Introduction

Alcohol abuse is a public health problem [1], [2], [3]. In Mexico, alcohol intake in the general population has increased over the years and young adults (18–29 years old) have the highest consumption, tending to a binge-drinking pattern [1]. Harmful patterns of drinking have shown to impact health, social behavior and immune function [2], [3], [4]. Several investigations have revealed an increased risk for cancer and infection in alcoholic subjects [2], [4] thus suggesting immune dysregulation induced by excessive alcohol use [5]. For example, chronically administrated alcohol reduces influenza A virus-specific T-CD8 quantity and their ability to degranulate and kill splenocytes pulsed with influenza peptides [6]. It has also been suggested that T-CD8 lymphocytes participate in liver damage, via nonspecific and antigen-specific mechanisms [7]. T-CD8 cells from cirrhotic mice transferred to severe combined immunodeficiency (SCID) mice induced liver fibrosis [8]. *In vitro* studies of human liver slices challenged with ethanol amounts equivalent to those of binge drinking showed that lymphocytes overexpressed the chemokine receptor CXCR4 and infiltrated the liver tissue [9]. Other chemokine receptors such as CCR5 [10] and CCR2 [11], [12] had also been implicated in liver inflammation and infiltration. On the other hand, peripheral lymphocyte activation was found in chronic alcoholics and correlated with total lifetime dose of ethanol consumed [13], also displaying higher cytotoxic activity [14]. Therefore, it is reasonable to suspect that the peripheral T-CD8 cells of binge drinkers might display altered expression of chemokine receptors as well as activation and cytotoxic molecules. However, this hypothesis has not been corroborated *in vivo*.

The aim of this study was to analyze the profile of peripheral T-CD8 in young adults according to alcohol consumption pattern (light, intermediate or binge) by analyzing the expression of a variety of molecules related to activating modulation (CD25, CD69, CD127, CD137, PD1, TLR4), trafficking (CCR2, CCR4, CCR5, CXCR4) and cytotoxic capacity (Granzyme B (GB)). Binge pattern showed a distinct T-CD8 profile with high CD69, low TLR4, CCR2 and CXCR4.

## Materials and Methods

### Study Participants

Protocol was approved by the Ethics Committee for Human Research of the Hospital General de México Dr. Eduardo Liceaga (Project No. DI/12/UME/04/007) and conducted in accordance with the ethical guidelines of the 1975 Declaration of Helsinki. Written consent for participation was obtained from patients. 55 participants were included, who self-reported as third generation Mexicans, age 18–29, free of known infectious disease (negative for HIV, HCV or HBV), abstained from psychotropic substance use, body mass index <30 kg/m^2^, non menstruating and otherwise healthy.

A 30ml sample of peripheral blood was collected via venipuncture to determine hepatic function; to screen for HIV, HBV and HCV presence; as well as for immunophenotyping assay. Participants were stratified in three patterns according to their alcohol consumption in three patterns determined by AUDIT (Alcohol Use Disorder Identification Test) [15] and HEPCA [16] surveys: Light, Intermediate and Binge. Light drinking pattern was defined as 1 or fewer drinks per day for women, 2 or fewer drinks per day for men, or abstention from alcohol for the last month, maintaining this habitual consumption. These criteria meet those of USD 2005 [17] for low-risk consumption. Intermediate drinking pattern was defined as a consumption of 2–4 drinks in more than two hours for women and 3–5 drinks in more than two hours for men, maintaining this habitual consumption. Binge drinking pattern was defined as 4 or more drinks in two hours for women and 5 or more drinks in two hours for men, maintaining this habitual consumption, according to NIAAA 2004 [18] criteria. Since HEPCA survey allows detailed analysis of consumption history, we were able to find that none of the participants in the Light group had heavy alcohol consumption history for more than 4 occasions and none of the participants in the Intermediate group had heavy alcohol consumption history for more than 1 year.

### Flow Cytometry

Peripheral leukocytes were analyzed by multifluorometric cytometry. Blood samples were placed on ice and delivered to the laboratory within 2 hours of collection to be processed with a commercial antibody according to manufacturer recommendation. For staining, leukocytes were obtained after erythrocyte lysis and resuspended in 300μl of PBS 1x. According to their fluorophores, 4 groups were established for staining with monoclonal antibodies: **group 1:** anti-CD3-FITC (OKT3), anti-CD8-PE-Cy7 (HIT8a), anti-CD25-PE (BC96), anti-CD69-APC-Cy7 (FN50), CD137-APC (4B4-1), anti-CD127-PerCP-Cy5.5 (A019D5), **group 2:** anti-CD3-APC-Cy7 (SK7), anti-CD8-PE-Cy7, anti-GB-FITC (GB11), anti-CXCR4-PE-Cy5 (12G5), anti-CCR4-Alexa 647 (TG6/CCR4), **group 3:** anti-CD3-APC-Cy7, anti-CD8-PE-Cy7, anti-TLR4-PE (HTA125), anti-CD137-APC, anti-GB-FITC, anti-CCR2-PE-Cy5 (TG5/CCR2), **group 4:** anti-CD3-APC-Cy7, anti-CD8-PE-Cy7, anti-CCR5-Alexa 647 (HEK/1/85a), anti-PD1-FITC (EH12.2H7), anti-CD127-PerCP-Cy5.5. All antibodies were acquired from Biolegend (San Diego, CA, USA) except anti-CD3-APC-Cy7 (BD Biosciences, San Jose, CA, USA).

Groups 1 and 4, leukocytes were fixed with 200 μl of paraformaldehyde 1% after incubation. Groups 2 and 3 were first stained with cell-surface antibodies, washed out after 20 minutes of incubation, fixed and permeabilized by Cytofix/Cytoperm Kit (BD Biosciences, San Diego, CA, USA) and anti-GB in Permwash (BD Biosciences, San Diego, CA, USA). Cells were washed out three times after overnight incubation and resuspended in PBS.

Samples were analyzed using BD FACSDiva software and a CANTO II flow cytometer (BD Biosciences, San Jose, CA, USA) with 2 lasers (488-nm and 640-nm). T-CD8 lymphocytes were identified by forward- and side-scatter (FSC and SSC), and presence of CD3 and CD8. 5x10^4^ T-CD8 cells were recorded. Results are shown as the percentage of positive cells and molecule expression is recorded as median fluorescence intensity (MFI). The instrument calibration was set in each experiment using single color–stained samples and limits were established using isotype and Fluorescence Minus One controls.

### Statistical Analysis

SPSS 20 (SPSS Inc., 2011) was used. Shapiro-Wilk test was used for normality and non-normal variables were transformed using log10, square root, square or 1/square root. Immunological variables were analyzed with one-way ANOVA tests. Levene tests were performed to determine homoscedasticity. DMS post-hoc test was used for homoscedastic variables and Tamhane for heteroscedastic variables. Chi-squared test was employed for gender. Size effect were calculated in variables with statistical difference and considered large when d≥0.8.

Factorial analysis was performed with principal components analysis (PCA) with VARIMAX rotation to identify the most prevalent grouping factors. The Kaiser–Meyer–Olkin (KMO) measure of sampling adequacy (cut-off 0.5), and Barlett’s Test of Sphericity (cut-off 0.001) were used to ensure the appropriateness of the data set for PCA. Cell populations were separated according to their immunological function into three main groups: activation, trafficking or cytotoxic capacity. One-way ANOVA tests were performed to compare these groupings with the different categories of alcohol consumption. Levene test and multi-comparison test were used as previously described. A *p*-value <0.05 was used for statistical significance. All data were reported as mean ± standard deviation. Prism 5 (GraphPad Software Inc, La Jolla, CA) was used to perform scatter plot graphs and Portable Statistica 8 (Statistica Software Inc, Tulsa, OK) was utilized for the XYZ factor analysis graph.

## Results

### Demographic and clinical data

All 55 subjects were grouped by alcohol consumption pattern. Recent alcohol consumption (2–6 days) was prevalent in the Binge group (88%) vs. Intermediate (55%) and Light (20%). AUDIT value increased with the amount of alcohol consumed. The Binge group consumed more drinks per occasion (12.6 ± 7.2) than both Intermediate (4.9 ± 2.5) and Light (3.2 ± 3.1) (p<0.01). Elevated Mean Corpuscular Volume (MCV) and γGT are commonly associated with excessive alcohol intake [19]. Binge group had no higher MCV but predictably, γGT was elevated compared to Light group (Table 1) although this elevation was allocated within the rank considered as clinically normal (less than 51U/L).

**Table 1:** Clinical and demographic characteristics of participants according to their drinking pattern.

Drinking pattern (n)	Light (20)	Intermediate (18)	Binge (17)	*p*
*Demographic*				
&Age (years)	22 ± 2.75^b^	24.39 ± 3.18^a^	25.12 ± 3.18^a^	<0.05
¥Gender F (%)	12 (60%)	9 (50%)	9 (53%)	NS
*Clinical*				
&BMI (kg/m^2^)	23.61 ± 2.34	24.2 ± 4.08	24.44 ± 2.63	NS
&Waist (cms)	79.39 ± 7.98	81.68 ± 10.46	84.31 ± 9.29	NS
&AUDIT	3.75 ± 3.56^c^	6 ± 3.88^b^	11.65 ± 6.4^a^	<0.05
€Drinks per occasion	3.2 ± 3.1^b^	4.9 ± 2.5^b^	12.6 ± 7.2^a^	<0.01
&ALT (U/L)	23.15 ± 15.09	21.61 ± 9.69	29.64 ± 26.74	NS
&AST (U/L)	24.5 ± 9.41	22.88 ± 8.4	25.17 ± 9.95	NS
&γGT (U/L)	16.95 ± 1.65^b^	21.33 ± 2.91^a^ ^,^ ^b^	34.94 ± 7.61^a^	<0.05
€MCV (fL)	92.29 ± 3.74	93.31 ± 3.82	77.45 ± 29.56	NS

^a,b,c^ Indicate homogeneous groups contrast, where a > b > c.

^¥^ Chi-squared Test.

^&^ One-way ANOVA DMS post-hoc test.

^€^ One-way ANOVA Tamhane post-hoc test.

### T-CD8 Profile

**Fig 1:**
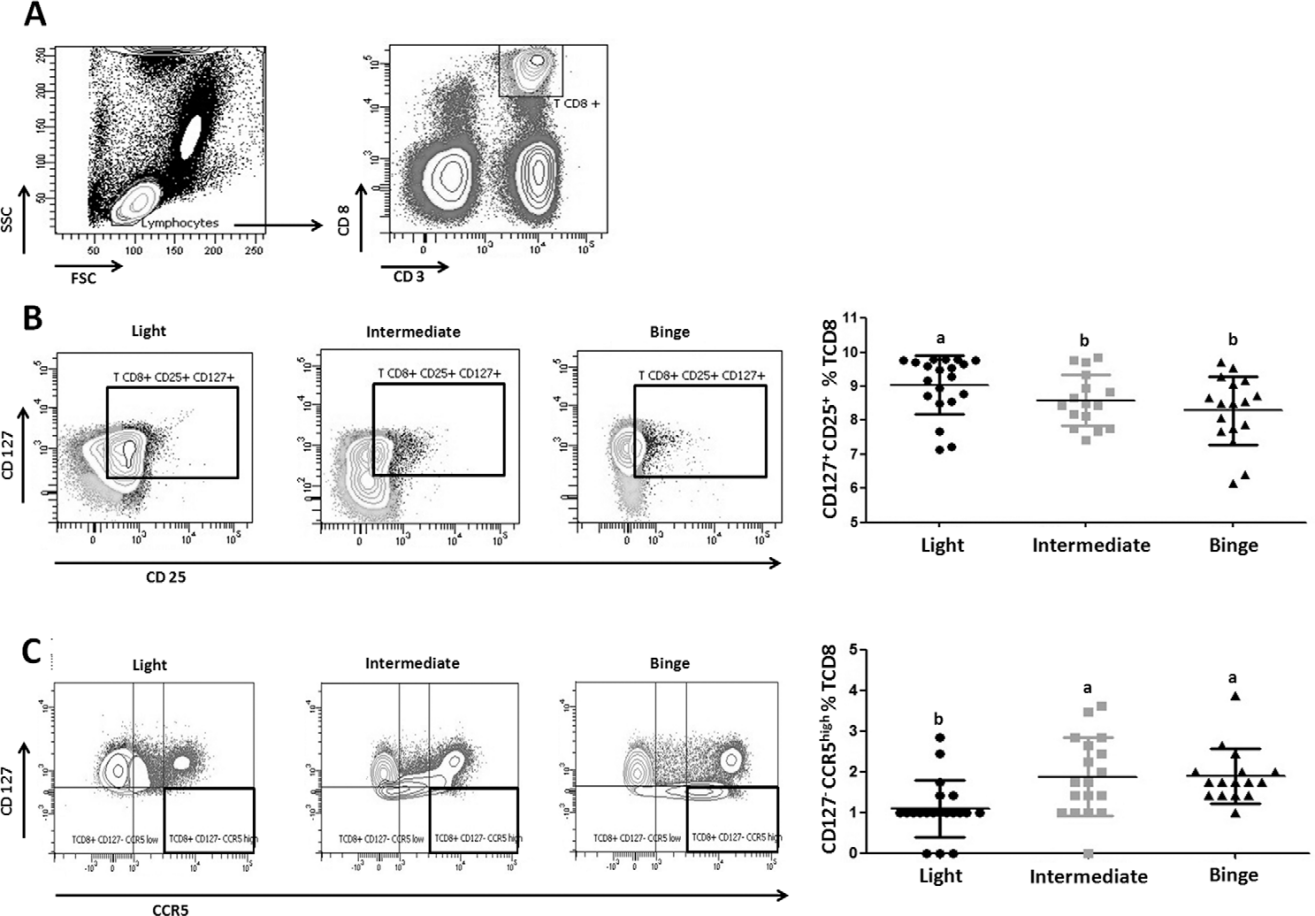
Alcohol consumption patterns modify the peripheral CD8 profile. **A)** Flow cytometry analysis of a representative sample of peripheral blood leukocytes; CD8 cells were sorted by forward vs. side scatter pattern (left panel) and the expression of CD3 and CD8 (right panel). Quadrants indicate percentage of CD8. Representative expression of the CD8 profile (left panel): CD25^+^CD127^+^ **(B)** and CD127^-^CCR5^high^ **(C)**. Scatter plot (right panel) summarizing the distribution (mean ± SD) of indicated CD8 phenotype according to alcohol consumption pattern: CD25^+^CD127^+^ **(B)** and CD127^-^CCR5^high^ **(C)**. ^a,b,c^Indicates homogeneous groups using DMS contrast where a > b > c.

T-CD8 lymphocytes were established by CD8 and CD3 expression (Fig 1A). According to the expression of different markers of immunological functions, T-CD8 profile was analyzed. CD69 is a marker of early activation with scarce expression in resting lymphocytes [20]. CD137 is only expressed on T-CD8 early after priming antigen, while being almost undetectable on resting cells, therefore is useful to detect and isolate antigen-specific T-CD8 [21]. CD127 is the alpha receptor for IL-7, a critical survival factor for lymphocytes by repressing the death factors Bad and Bax [22]. CD25 is the alpha receptor for IL-2, and combined with CD127 allows the identification of conventional and regulatory T cells [23], [24]. PD1 (Programed death protein 1), an inhibitory receptor, is often associated with cellular exhaustion and was recently associated with acute alcoholic hepatitis [25]. TLR4 (Toll-like receptor 4) recognizes LPS and is significantly involved in the alcoholic liver fibrosis [26].

**Fig 2:**
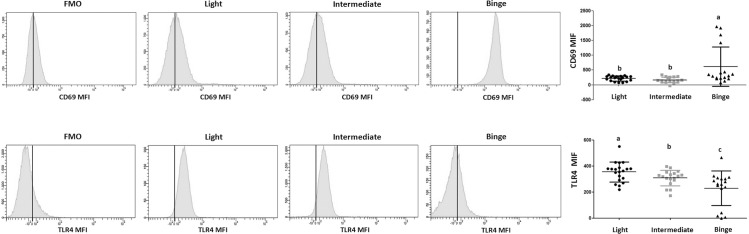
Alcohol consumption patterns modify the expression of CD69 and TLR4 in peripheral CD8. Representative FACS histograms for MFI of CD69 (upper panel) and TLR4 (lower panel) in peripheral CD8 population. MFI comparison of control (as FMO) and alcohol consumption groups (left panel). Scatter plot (right panel) summarizing the MFI of CD69 (upper panel) and TLR4 (lower panel) separated by alcohol consumption pattern. FMO: fluorescence minus one. ^a,b,c^Indicates homogeneous groups using Tamhane contrast where a > b > c.

Regarding *Activation*, the percentage of T-CD8 CD25^+^ CD127^+^ is lower in Binge drinkers when compared to Light drinkers, while the percentage of T-CD8 CD127^-^ CCR5^high^ is higher in the Binge group (Fig 1B and 1C). Also, the Binge group presented higher expression of CD69 (recent activation) and lower expression of both TLR4 (sensitivity to LPS), PD1 (down regulator) and percentage of T-CD8 CD137^+^ (antigen-specific activation) (Fig 2, Table 2).

**Table 2:** Immunophenotyping of peripheral T-CD8 participants according to their drinking pattern.

Drinking pattern (n)	Light (20)	Intermediate (18)	Binge (17)	*p*
Immunophenotype:				
***Activation***				
&PD1 MFI ON T-CD8	2.45 ± 0.16^a^	2.32 ± 0.18^b^	2.32 ± 0.17^b^	<0.05
^€^CD69 MFI ON T-CD8	218.40 ± 85.47^b^	163.16 ± 95.64^b^	618.11 ± 664.21^a^	<0.05
€TLR4 MFI ON T-CD8	254.85 ± 76.61^a^	208.50 ± 59.12^b^	129.05 ± 132.72^c^	<0.01
^&^ % T-CD8 CD137^+^	1.34 ± 0.40^a^	1.35 ± 0.41^a^	1.00 ± 0.30^b^	<0.05
& % T-CD8 TLR4^+^	2.35 ± 1.11^a^	1.91 ± 0.67^a^	1.20 ± 1.06^b^	<0.05
^€^ % T-CD8 PD1^+^ CD127^+^	3.63 ± 1.53^a^	2.37 ± 1.12^b^	2.28 ± 0.98^b^	<0.05
€ % T-CD8 CD127^+^ CD137^+^	1.45 ± 1.63^a^	0.22 ± 0.73^b^	0.23 ± 0.56^b^	<0.05
^&^ % T-CD8 CD25^+^ CD127^+^	1.62 ± 0.21^a^	1.41 ± 0.18^b^	1.36 ± 0.24^b^	<0.01
€ CD69 MFI ON T-CD8 CD25^+^ CD137^+^	3.08 ± 0.38^b^	3.46 ± 0.19^a^	3.42 ± 0.46^a^ ^,b^	<0.01
^&^ CD69 MFI ON T-CD8 CD127^+^ CD137^+^	0.04 ± 0.01^a^	0.03 ± 0.008^b^	0.02 ± 0.001^b^	<0.01
***Trafficking***				
^&^ % T-CD8 CXCR4^+^ CCR4^-^	9.03 ± 0.85^a^	8.57 ± 0.74^a^ ^,b^	8.28 ± 1.00^b^	<0.05
& % T-CD8 CD127^-^ CCR5^high^	1.09 ± 0.69^b^	1.87 ± 0.95^a^	1.89 ± 0.66^a^	<0.01
^&^CCR2 MFI ON T-CD8	465.90 ± 217.11^a^	483.05 ± 137.79^a^	286.94 ± 157.78^b^	<0.01
&CXCR4 MFI ON T-CD8	3.51 ± 0.21^a^	3.31 ± 0.18^b^	3.30 ± 0.20^b^	<0.01
***Effector Capacity***				
& % T-CD8 Granzyme B^+^ CXCR4^-^	1.09 ± 1.39^b^	2.16 ± 1.32^a^	2.48 ± 1.45^a^	<0.05
*Trafficking and Effector Capacity*				
€ CXCR4 MFI ON T-CD8 Granzyme B^+^ CXCR4^+^	3.23 ± 0.18^a^	3.11 ± 0.12^a^ ^,^ ^b^	3.09 ± 0.11^b^	<0.01
***Activation and Trafficking***				
& % T-CD8 PD1^+^ CCR5^+^	4.20 ± 1.33^a^	3.46 ± 1.07^a^ ^,^ ^b^	3.08 ± 1.30^b^	<0.01
^&^ % T-CD8 PD1^+^ CCR5^-^	0.92 ± 0.23^a^	0.61 ± 0.26^b^	0.63 ± 0.28^b^	<0.01
€ % T-CD8 CD127^+^ CCR5^low^	1.04 ± 0.33^a^	0.99 ± 0.20^a^	0.70 ± 0.29^b^	<0.01
*Activation*, *Trafficking and Effector Capacity*				
& TLR4 MFI ON T-CD8 Granzyme B^+^ CCR2^+^	477.90 ± 280.54^a^ ^,^ ^b^	607.50 ± 290.33^a^	268.88 ± 420.10^b^	<0.01

^a,b,c^ Indicate homogeneous groups contrast, where a > b > c.

^&^One-way ANOVA DMS post-hoc test.

^€^One-way ANOVA Tamhane post-hoc test.

In regard to *Trafficking*, the Binge group had higher percentage of T-CD8 CD127^+^CCR5^high^ and lower expression of CCR2 and CXCR4 on T-CD8. The Binge group also had high percentage of T-CD8^+^GB^+^CXCR4^+^ (Table 2).

### Factorial analysis

Since the immune system works as an integrative mechanism by combining different sets of cellular phenotypes, and in order to associate clusters of these phenotypes, a factor analysis was performed. Only molecular expression on T-CD8 by MFI was used for PCA. VARIMAX rotation showed 3 components with high factor loading. These components were labeled Early Activation, Effector Activation and Trafficking (Table 3). The KMO measure (Activation: 0.547 and Trafficking: 0.685) and Bartlett’s Test of Sphericity (*p*<0.001 in both groups) indicated sampling adequacy and variability, respectively.

**Table 3:** Measure loadings after varimax rotation of first three components of principal component analysis. Rotated factor loadings variables of activation, trafficking and cytotoxicity settings were used to conform the components as described in Material and Methods.

Variable	Eigenvalues
**Early Activation**	
CD69 MIF ON T CD8	**-0.953**
TLR4 MFI ON T CD8	**0.845**
**Effector Activation**	
CD69 MFI ON T CD8 CD127^+^ CD137^+^	**0.915**
CD69 MFI ON T CD8 CD25^+^ CD137^+^	**-0.958**
**Trafficking**	
CXCR4 MFI ON T CD8	**0.733**
CXCR4 MFI ON T CD8 Granzyme B^+^ CXCR4^+^	**0.742**
CCR2 MFI ON T CD8	**0.808**

**Fig 3:**
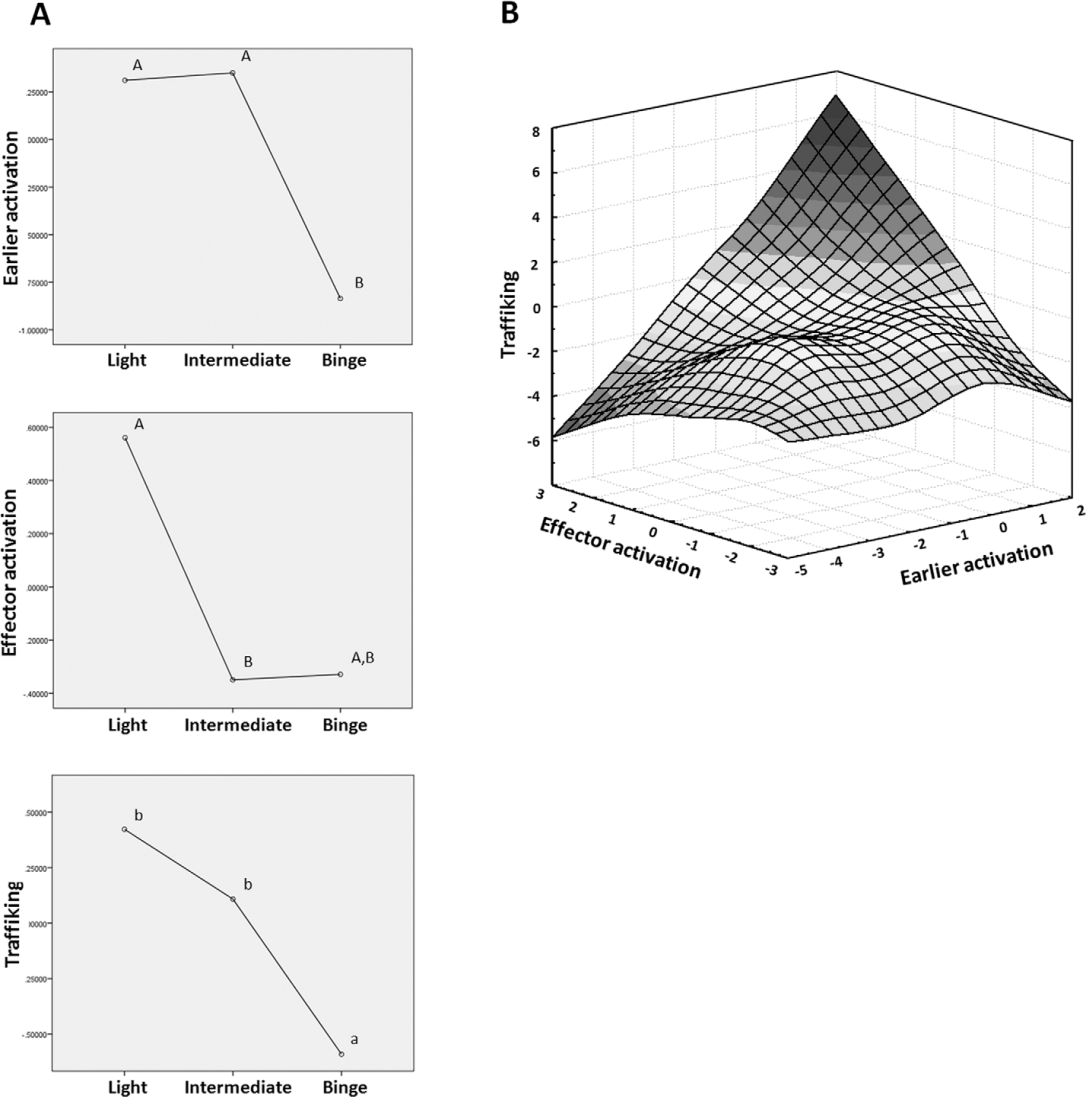
Factorial analysis. **A)** One way ANOVA stratified by alcohol consumption pattern and 3 specific factors: Early Activation (Upper graph), Effector Activation (Middle graph) and Trafficking (Lower graph) **B)** Association of the factors in a XYZ surface plot, Z axis Trafficking factor, X axis Early Activation factor and Y axis Effector Activation factor. ^a,b,c^Indicates homogeneous groups using DMS contrast where a > b > c. ^A,B,C^Indicates homogeneous groups using Tamhane contrast where A > B > C.

The Binge group exhibited fewer Early Activation Factor (Fig 3A up) and Trafficking Factor (Fig 3A down) than the Light and Intermediate groups. The Intermediate group shows fewer Effector Activation factors than the Light group (Fig 3A middle). The Light group exhibits the highest levels ​(Fig 3B, dark area) while the Binge group exhibits the lowest.

## Discussion

The AUDIT questionnaire was developed to screen excessive drinking patterns and help practitioners to identify people who would benefit from reduction or abstention from drinking [27]. Although widely used in medical practice, this scale does not take in consideration the rapidity of consumption or alcohol type as the HEPCA survey does [16]. Light, Intermediate and Binge groups have AUDIT indexes concordant with increasing consumption. The Intermediate group has a level of consumption between low-risk and hazardous drinking, and their AUDIT index average is around 8. γGT level is customarily used as a marker for heavy alcohol use [28] and alcoholic liver disease [29]; unsurprisingly this marker is increased in the Binge drinking group when compared to the Light-drinking group (Table 1).

Numerous immunological changes by heavy alcohol consumption have been identified. Some examples of those are: Activated alveolar macrophages, plasma levels of cytokines increased [30], decreased frequency of LPS-specific mononuclear cells producing IFN-gamma [25], activated iNKT [31], decreased density of dendritic epidermal T cells [32] and reduced cytotoxic capacity of CD8 T cells [6].

T-CD8 lymphocytes are a remarkable subpopulation that has the ability to modulate their environment through cytokine secretion and direct cytotoxicity [33]. Laso et al. [14], found increased numbers and cytotoxic activity of T-CD8 cells in individuals with chronic alcohol consumption without liver disease. Furthermore, Jiménez-Ortega et al. [34], showed in peripubertal rats that discontinuous drinking of moderate amounts of ethanol can alter the immune system more severely than continuous ethanol intake, as measured by decreased lymph node and splenic T-CD8^+^ cells. This suggests that peripheral T-CD8 cells may have altered profiles of activation, trafficking and cytotoxic capacity in young individuals consuming alcohol in a binge drinking pattern.

CD69 is a membrane receptor transiently expressed in lymphocytes, including T-CD8 [35], associated with early leukocyte activation and closely related to inflammation [36]. Zhang & Meadows [37] showed that chronic alcohol consumption in murine models does not alter the percentage of CD69^+^ in T-CD8, but the expression was not analyzed further. They did observe an increase in T-CD8^+^Ly6C^+^CD44^int/hi^ and Interferon-gamma (IFN-γ) producing T-CD8 lymphocytes, which may mean that this cell subtype has a constant activation pattern. Early studies in alcoholic adults showed increase in peripheral blood lymphocytes expressing CD69 and CD25 [13]. The Binge drinking group exhibited increased CD69 expression in peripheral T-CD8, which could represent early activation.

Hepatic fibrogenesis requires the interaction of several main factors: hepatic injury induced by alcohol itself and a defective intestinal barrier, an increased intestinal bacterial microbiota, functional TLR4 and hepatic exposure to bacterial lipopolysaccharide (LPS) [38]. TLR4 knockout mice fed alcohol did not develop liver damage [39] and TLR4 expression in bone marrow derived cells is needed for fibrogenic response to chronic alcohol consumption [40]. We found a decrease in expression of TLR4 on T-CD8 in the Intermediate drinking group and a further decrease in the Binge drinking group (Fig 2). Although TLR4 has been mainly associated with cells of the innate immune system, expression on T-CD8 cells also recognizes LPS and leads to the release of IFN-γ and GB [41], which is part of a fast-response modulatory mechanism. This deficiency in the Binge drinking group could be associated with a higher prevalence of infections [42].

GB, one of the main components of lytic granules, is able to cleave caspases. In animal models, chronic alcohol consumption modifies splenic GB [43]. Our results show increases in the percentage of peripheral T-CD8^+^GB^+^CXCR4^-^ in the Intermediate and Binge drinking groups (Table 2). This type of cell has cytotoxic potential but is not sensitive to CXCL12. Given that GB is a cytotoxic molecule, the increase in this phenotype (T-CD8^+^GB^+^CXCR4^-^) could be involved in contributing to alcoholic liver disease.

Chemokines orchestrate leukocyte recruitment in tissues and their immunological influence is a combination of the modulations made by the cells they affect. Chemokines mediate other biological activities and a single chemokine may be active in a number of cell populations [44]. Our results showed an increase in the percentage of T-CD8^+^CDC127^-^CCR5^high^ phenotype in the Intermediate and Binge drinking patterns of consumption vs. Light drinking (Table 2). Moreno et al. [10] highlighted the role of CCR5 in immune response as a governing fact that limits liver inflammation. They found that liver injury is severely worsened in CCR5 knockout mice by inducing an increase in production of CCL3, CCL4 and CCL5, thereby enhancing recruitment of inflammatory cells into the liver and production of proinflammatory cytokines. Following this logic, the increased percentage of T-CD8^+^CCR5^high^ in the Intermediate and Binge drinking groups may be a reaction aimed at regulating damage from alcohol. It would be necessary to assess whether these cells have a regulatory rather than effector role in future studies.

Binge drinking of alcohol is correlated with a decrease in Early Activation factors (Fig 3A). Because of the negative association of CD69 in the PCA (Table 3), its expression level in peripheral blood T-CD8 is increased in proportion to the decrease of TLR4. This reflects an early activation of T-CD8 (defined by higher CD69) accompanied by insensitivity to LPS or endogenous ligands such as HMGB1 [35] (defined by lower TLR4). This could be a result of intermittent stimulation of the same TLR4 that is internalized and diminished on the cell surface. Moreover, the Effector Activation factor is integrated by the expression of CD69 on T-CD8^+^CD127^+^CD137^+^ and on T-CD8^+^CD25^+^CD137^+^ (Table 3). T-CD8 CD137^+^ cells are specifically antigen-activated [45] which is not necessarily the case with CD25^+^ cells, which could be T regulator cells [46]. It is also known that T-CD8 cells expressing CD127 have higher survival rates [47]. This Effector Activation factor is decreased in Intermediate and Binge drinking groups (Fig 3A) and could represent a peripheral and perhaps quite significant regulatory component. Future research into markers of T reg such as Foxp3 is necessary to investigate this possibility.

We found the Trafficking factor decreased in the Binge drinking group compared with the Intermediate and Light drinking groups (Fig 3A). Karim et al. [9] showed in an *in vitro* model that lymphocytes exposed to binge drinking levels of alcohol overexpress CXCR4 and infiltrate the liver. Our results show a decrease in the expression of CXCR4 in T-CD8 lymphocytes of peripheral blood. This may suggest a sequestration of T-CD8 with high expression of CXCR4 in the liver. Several researchers [48], [49], [50], [51], [52], [53] have shown the relationship between large quantities of alcohol consumption and the generation of protein adducts, which function as neo-antigens and induce an immunological response. Most of the cells expressing CXCR4 in peripheral blood are naïve [54]; when these cells acquire effector capacity the levels of CXCR4 tend to diminish [55]. The diminished CXCR4 in peripheral lymphocytes in our study suggests a switch in effector capacity in response to neo-antigens generated by protein adducts as a result of alcohol metabolism; a possibility that is consistent with the work of Kobayashi [55] and Karim [9]. The strong CXCR4 expression data in our study may be due to the fact that CXC chemokines are more powerful than CC chemokines, and SDF-1 (ligand of CXCR4) is stronger than either chemokine [54]. Furthermore, CCR2 deficiency reduces liver inflammation and fibrosis [11] and its ligand MCP-1 is known to mediate inflammatory cell activation in the liver [12]. In our study, CCR2 expression on T-CD8 is diminished in the Binge drinking group, which could be associated to mobility to the liver.

The associations between the three immunophenotypic groupings (Fig 3B) show that the factors within each of the Early Activation, Effector Activation and Trafficking cohorts increase and diminish in a joint manner. When the Early Activation factors decrease, a small elevation seems to form before completely decreasing, likely representing a transitional group composed of the Binge drinking subjects. In contrast, the Light drinking group subjects have very elevated values. When the Effector Activation factors increase, the Trafficking and Early Activation factors tend to diminish in a linear relationship similar to the Early Activation factors including the notable transitional group likely composed of the Intermediate group subjects in this case. Given these trends, it seems that alcohol consumption patterns induce distinct immune responses.

In summary, our results showed that alcohol consumption modifies the peripheral T-CD8 cell profile in human participants. In the Activation immunophenotype, CD69 and TLR4 showed opposite effects with TLR4 decreasing as CD69 increased in the Binge drinking group; the Trafficking immunophenotype showed a decrease in the expression of CXCR4, CCR2 on T-CD8 exclusively in the Binge-drinking group, while CCR5^high^ showed an increase in the Intermediate- and Binge- drinking groups compared with the Light-drinking group. The cytotoxic capacity immunophenotype was increased in the Intermediate- and Binge-drinking groups compared to the Light-drinking group. Therefore, we can conclude that decreased expression of Early Activation and Trafficking factors are specific to the binge-drinking pattern of alcohol consumption while elevated expression of Effector Activation factors is associated with a light-drinking pattern of alcohol consumption. The immunologic modulation caused by excess alcohol consumption may lead to an increased susceptibility to T-CD8 mediated infections such as HCV or influenza as reported in other studies. To our knowledge, this is the first study that links activation, trafficking and cytotoxic capacity in peripheral T-CD8 to alcohol consumption. Further research into this newly discovered immune profile is necessary in order to further describe the relationship between the immune system and liver damage.
